# Seawater Immersion Hypothermia Triggers Cardiac Pyroptosis via the NF-κB/NLRP3 Inflammasome Axis: A Mechanistic Study in Rats

**DOI:** 10.3390/ijms27135890

**Published:** 2026-06-30

**Authors:** Huifang Deng, Chaoyue Sun, Zhibo Wang, Hongbiao Chen, Yiwen Ben, Yukun Wu, Wumu Xu, Jiaqi Wang, Yajing Wang, Yanrong Gong, Yunyang Wu, Xiaofei Zhu, Wei Gu, Zifei Yin

**Affiliations:** 1 School of Traditional Chinese Medicine, Naval Medical University, Shanghai 200433, China; 2 Basic Medicine College, Naval Medical University, Shanghai 200433, China; 3 Graduate School, Naval Medical University, Shanghai 200433, China; 4 Changhai Hospital, Naval Medical University, Shanghai 200433, China

**Keywords:** seawater immersion, hypothermia, cardiac injury, NLRP3 inflammasome, pyroptosis

## Abstract

Cold seawater immersion is a critical lethal risk in maritime accidents and military operations, frequently inducing fatal myocardial dysfunction. However, the mechanisms underlying this seawater immersion hypothermia-induced cardiac injury remain poorly defined. This study aimed to elucidate the pathological progression and underlying mechanisms of myocardial injury induced by cold seawater immersion. A male SD rat model was immersed in 15 °C seawater for 2 h. Echocardiography, transmission electron microscopy, transcriptomics, and Western blot were performed to assess cardiac function, mitochondrial ultrastructure, and molecular mechanisms. Cold stress triggered progressive bradycardia (~480 to ~100 bpm) with initial Frank–Starling compensation, followed by decompensation with reduced cardiac output and impaired diastolic function. Mitochondrial ultrastructural damage preceded histological lesions and was accompanied by elevated cardiac injury markers (cTnT, CK-MB, BNP). Cardiac tissue exhibited upregulated TNF-α, IL-1β, and IL-6, while transcriptomic analysis revealed enrichment of inflammatory pathways (TNF, NF-κB) and coordinated upregulation of pattern recognition receptors including scavenger receptor, Toll-like receptor, and NOD-like receptor families. The Western blot confirmed NF-κB activation, NLRP3 inflammasome assembly, and the N-terminal fragment of gasdermin D (GSDMD-NT) accumulation, indicating pyroptotic cell death. These findings demonstrate that cold seawater stress disrupts mitochondrial homeostasis and activates the NF-κB/NLRP3/pyroptosis cascade, contributing to inflammatory cardiomyocyte death and cardiac decompensation. This mechanistic insight may inform therapeutic strategies for seawater immersion hypothermia.

## 1. Introduction

Maritime emergencies remain frequent globally, posing a significant threat to human life despite ongoing advances in navigation safety and regulatory frameworks. According to the European Maritime Safety Agency (EMSA), over 2600 marine casualties occurred annually between 2015 and 2024, resulting in substantial fatalities and injuries [1]. Research by the Maritime Accident Investigation Branch (MAIB) has shown that more than 40% of man overboard occurrences between 2015 and 2023 tragically led to a fatality [2]. Once a person falls into cold water below 15 °C, the physiological response progresses through four stages: cold shock (0–3 min), incapacitation (10–15 min), hypothermia (>30 min), and circum-rescue collapse [3]. The first two stages kill primarily through drowning. Sudden immersion triggers gasping and tachycardia, rapidly compromising swimming ability and leading to water aspiration [4]. Drowning is therefore traditionally regarded as the leading cause of immersion fatalities. However, if the victim remains afloat, hypothermia becomes the primary threat [5]. Maritime epidemiological data indicate that roughly 10–14% of seawater immersion victims present hypothermic manifestations [6,7]. With progressive hypothermia, core temperature drops rapidly. The heart, a high-metabolism organ, becomes functionally suppressed, leading to a range of serious physiological disturbances such as arrhythmias, decreased cardiac output, and a lowered threshold for ventricular fibrillation [8]. Over 36% of patients with severe accidental hypothermia may develop hypothermic cardiac arrest (“rescue collapse”), with a high mortality rate of 64% [9]. While the first two stages claim lives within minutes, the hypothermic stage unfolds over a longer time window and involves progressive cardiac dysfunction that may be amenable to intervention. Thus, elucidating the pathological mechanisms underlying seawater immersion hypothermia-induced cardiac injury is of significant practical and military importance.

The detrimental impact of hypothermia on cardiac function spans the entire continuum from cooling to rewarming. Core cooling triggers peripheral vasoconstriction, increasing cardiac afterload and impairing coronary perfusion [10]. Moderate hypothermia frequently causes pro-arrhythmic changes in cardiac electrophysiology [11]. Deep hypothermia reduces cardiac output and oxyhemoglobin saturation, thereby limiting systemic oxygen delivery and impairing tissue offloading, which exacerbates myocardial ischemia and hypoxia [12,13,14]. During rewarming, the risk of cardiovascular compromise may increase [8]. Previous studies have implicated mitochondrial dysfunction, apoptosis, autophagy, oxidative stress, and inflammatory pathways in cold stress- and rewarming-induced myocardium injury [15,16,17]. However, most current studies on cold stress-induced myocardial injury focus on cold air exposure, freshwater immersion, or therapeutic hypothermia. Cold seawater immersion differs from these conditions by its rapid heat extraction (water conducts heat 25 times faster than air), sustained wet cold stimulation, and additional hyperosmotic stress, triggering more rapid physiological deterioration and shorter survival duration than freshwater under equivalent low-temperature conditions [18]. Whether these unique features exacerbate cardiac injury through distinct molecular pathways remains unclear.

Compelling evidence has established that cold stress strongly interfaces with inflammatory cascades, driving myocardial damage via inflammation, oxidative stress, and pyroptosis [19,20,21]. The nuclear factor-kappaB (NF-κB) signalling pathway, activated by various stimuli including cold stress, serves as a central regulator of cellular immune responses in physiology and disease [22]. NF-κB activation is a prerequisite for the transcriptional priming of NLR family pyrin domain containing 3 (NLRP3) and pro-interleukin-1β (pro-IL-1β) and pro-interleukin-18 (pro-IL-18) [23]. The assembled NLRP3 inflammasome cleaves pro-caspase-1 into active caspase-1, which then processes pro-IL-1β and pro-IL-18 into their mature, highly active forms, triggering pyroptosis, a pro-inflammatory form of programmed cell death, and fueling a local inflammatory cascade that exacerbates tissue injury [24]. Findings from pig and rodent models showed that cold stress activated the NLRP3 inflammasome, leading to lung injury through pyroptosis [25,26]. Recent study reported that in rats subjected to 15 °C seawater immersion, IL-1β was the most significantly upregulated cytokine in both intestinal tissue and circulation, and its elevation was effectively mitigated by rewarming [27], highlighting the importance of the NLRP3/IL-1β axis in seawater immersion hypothermia. Despite these advances, a significant knowledge gap persists regarding the role of this specific inflammatory axis in cardiac injury induced by seawater immersion hypothermia.

Here, we aimed to investigate the pathological progression and molecular mechanisms underlying seawater immersion hypothermia-induced cardiac injury. Using a rat model of 15 °C seawater immersion, we sought to characterize the temporal progression of myocardial dysfunction and inflammatory responses. Bulk RNA sequencing was employed to identify dysregulated signaling pathways, and molecular assays were performed to validate potential mechanisms. By elucidating the initiating events during sustained hypothermia, this study may provide a foundation for understanding hypothermia-related cardiac damage and may inform therapeutic strategies for seawater hypothermia resuscitation.

## 2. Results

### 2.1. Cold Seawater Immersion Impaired Cardiac Function and Induced Compensatory Remodeling in Rats

**Figure 1:**
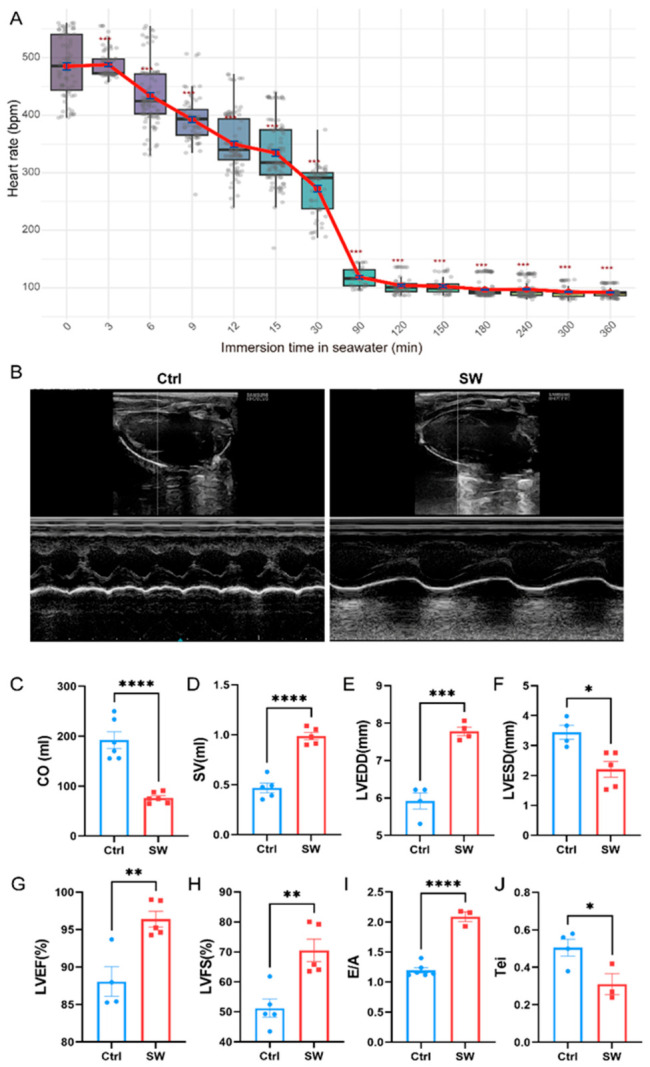
Cold seawater immersion induces severe bradycardia and adaptive cardiac remodeling with impaired global function in rats. (**A**) Time-course analysis of heart rate (HR) during 15 °C seawater immersion (*n* = 8 per time point). (**B**) Representative M-mode echocardiographic images of left ventricular (LV) morphology in control (Ctrl) and seawater-immersed (SW) rats at the end of 2 h immersion (*n* = 6). (**C**–**J**) Quantitative echocardiographic parameters at 2 h: (**C**) cardiac output (CO), (**D**) stroke volume (SV), (**E**) LV end-diastolic diameter (LVEDD), (**F**) LV end-systolic diameter (LVESD), (**G**) LV ejection fraction (LVEF), (**H**) LV fractional shortening (LVFS), (**I**) E/A ratio (diastolic filling), and (**J**) Tei index (global myocardial performance). Data are presented as box plots with individual data points; * *p* < 0.05, ** *p* < 0.01, *** *p* < 0.001, and **** *p* < 0.0001.

To evaluate the effects of hypothermic (15 °C) seawater immersion on cardiac function and structure, rats were subjected to continuous electrocardiogram (ECG) monitoring and echocardiography at 2 h. Immersion induced progressive bradycardia: heart rate progressively declined from a baseline of ~480 bpm to ~260 bpm within the first 30 min, dropping sharply to ~120 bpm at 90 min, and stabilizing at ~100 bpm by 120 min (Figure 1A). M-mode echocardiography at 2 h revealed ventricular cavity dilation and markedly reduced wall motion in the seawater-immersed (SW) group compared to the regular rhythm and robust contraction of the controls (Ctrl) (Figure 1B). Quantitative analysis showed that while stroke volume (SV) was significantly increased in the SW group (*p* < 0.0001, Figure 1D), cardiac output (CO) was markedly decreased (*p* < 0.0001, Figure 1C), a phenotype attributable to severe immersion-induced bradycardia. This bradycardic state was accompanied by significant ventricular remodeling, including increased left ventricular end-diastolic diameter (LVEDD, *p* < 0.001, Figure 1E) and decreased left ventricular end-systolic diameter (LVESD, *p* < 0.05, Figure 1F), indicating compensatory preload recruitment. Consequently, both left ventricular ejection fraction (LVEF, *p* < 0.01, Figure 1G) and fractional shortening (LVFS, *p* < 0.01, Figure 1H) were significantly elevated, reflecting a Frank–Starling-mediated compensatory response to hypoperfusion. However, diastolic function was compromised, as evidenced by a significantly increased E/A ratio (*p* < 0.0001, Figure 1I), suggesting restrictive filling or decreased compliance. Global myocardial efficiency, as assessed by the Tei index, was significantly reduced (*p* < 0.05, Figure 1J) in the SW group. This reduction, driven by a compensatory prolongation of ejection time in the setting of severe bradycardia, reflects an adaptive mechanism to maintain stroke output. In summary, 2 h of cold seawater immersion induced a state of severe bradycardia with compensatory high stroke output. This compensation, however, was insufficient to prevent a significant, heart rate-dependent decline in cardiac output, culminating in circulatory failure.

### 2.2. Cold Seawater Immersion Induced Myocardial Injury and Mitochondrial Damage in Rats

**Figure 2:**
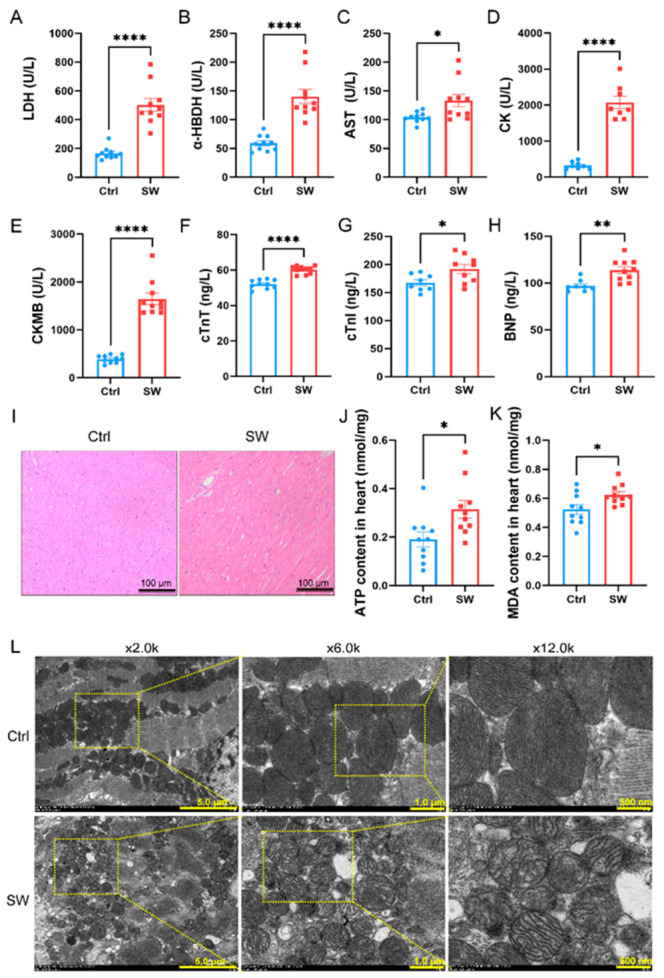
Cold seawater immersion triggers severe myocardial injury and mitochondrial damage in rats. (**A**–**H**) Plasma levels of myocardial injury and cardiac dysfunction biomarkers in control (Ctrl) and seawater-immersed (SW) rats at the end of 2 h immersion: (**A**) lactate dehydrogenase (LDH), (**B**) α-hydroxybutyrate dehydrogenase (α-HBDH), (**C**) aspartate transaminase (AST), (**D**) creatine kinase (CK), (**E**) creatine kinase-MB (CK-MB), (**F**) cardiac troponin T (cTnT), (**G**) cardiac troponin I (cTnI), and (**H**) B-type natriuretic peptide (BNP). Data are presented as box plots with individual data points (*n* = 10). (**I**) Representative hematoxylin and eosin (H&E) staining images of myocardium at the end of 2 h immersion (*n* = 6, scale bar: 100 μm). (**J**,**K**) Myocardial ATP and MDA content at the end of 2 h immersion, normalized to total protein (*n* = 10). (**L**) Representative transmission electron microscopy (TEM) images of left ventricular mitochondria at the end of 2 h immersion. (*n* = 3, scale bars: 5.0 μm, 1.0 μm, and 500 nm, as indicated). * *p* < 0.05, ** *p* < 0.01, and **** *p* < 0.0001.

To assess the effects of cold seawater immersion on myocardial injury and subcellular structure, plasma samples were collected for biochemical analysis and cardiac tissue was examined by transmission electron microscopy (TEM) at the end of 2 h immersion. Compared with the Ctrl group, the SW group exhibited significantly elevated levels of multiple plasma markers of myocardial injury, including lactate dehydrogenase (LDH, *p* < 0.0001, Figure 2A), α-hydroxybutyrate dehydrogenase (α-HBDH, *p* < 0.0001, Figure 2B), aspartate transaminase (AST, *p* < 0.05, Figure 2C), and creatine kinase (CK, *p* < 0.0001, Figure 2D), indicating widespread cellular damage. More cardiac-specific markers, including creatine kinase-MB (CK-MB, *p* < 0.0001, Figure 2E), cardiac troponin T (cTnT, *p* < 0.0001, Figure 2F), and cardiac troponin I (cTnI, *p* < 0.05, Figure 2G), were also markedly increased in the SW group. Furthermore, plasma B-type natriuretic peptide (BNP, *p* < 0.01, Figure 2H), a biomarker of cardiac load and heart failure, was significantly elevated, further confirming the presence of cardiac dysfunction.

Consistent with these biochemical changes, H&E staining (Figure 2I) showed clear morphological alterations in the myocardium of SW rats, including disordered myofibrillar arrangement, cellular swelling, and increased interstitial space, compared with the well-organized structure in the Ctrl group. Functionally, myocardial ATP content was significantly elevated (*p* < 0.05, Figure 2J) in the SW group, whereas malondialdehyde (MDA) levels, a critical indicator of lipid peroxidation, were also markedly increased (*p* < 0.05, Figure 2K). These changes indicated abnormal energy metabolism compensation and excessive oxidative stress following cold seawater immersion. TEM imaging (Figure 2L) revealed well-preserved mitochondria in control hearts, characterized by regular oval shapes, densely packed and parallel cristae, and homogeneous matrix density. In contrast, mitochondria from SW hearts were smaller, more rounded, and more numerous, and exhibited obvious ultrastructural damage, including marked swelling, irregular morphology, disorganized, sparse, or fractured cristae, prominent vacuolization, and reduced matrix density. Collectively, these results demonstrated that 2 h of cold seawater immersion induced profound myocardial enzyme leakage and subcellular damage, which may be closely associated with mitochondrial dysfunction and oxidative stress.

### 2.3. Systemic and Cardiac Inflammatory Response Following Cold Seawater Immersion

**Figure 3:**
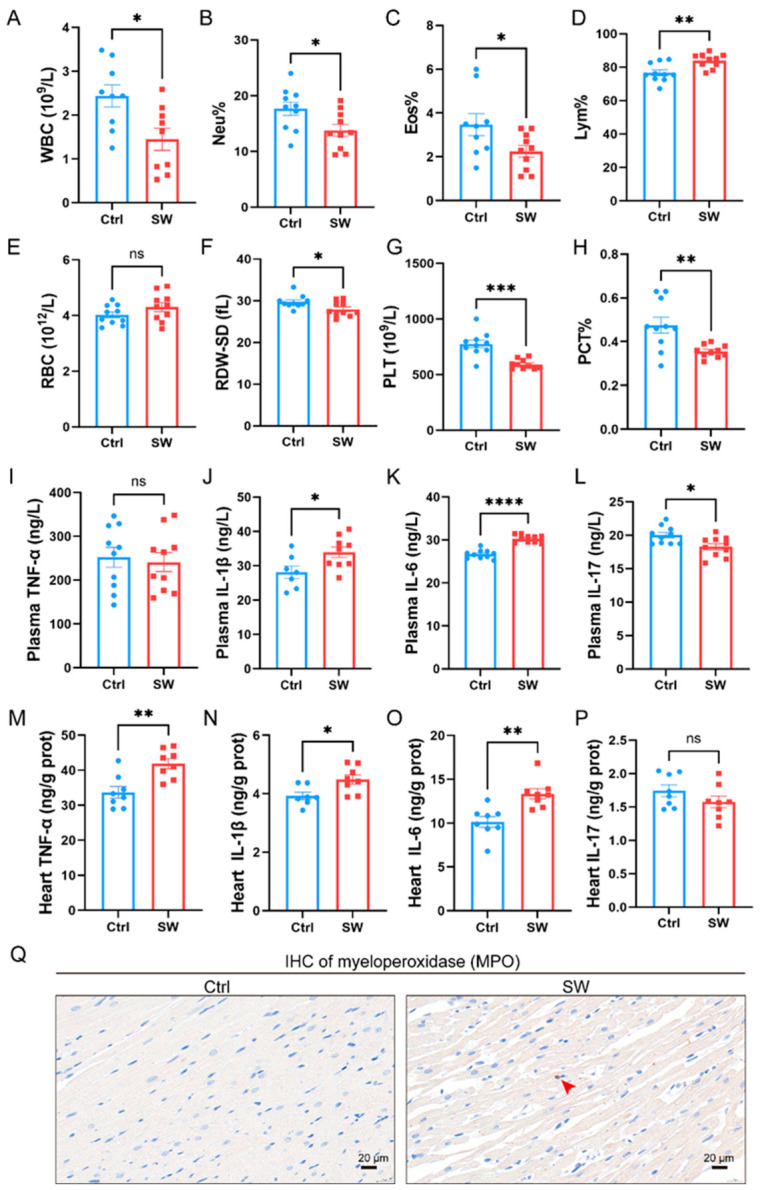
Cold seawater immersion induces systemic hematological alterations and local myocardial inflammation in rats. (**A**–**H**) Hematological parameters in control (Ctrl) and seawater-immersed (SW) rats at the end of 2 h immersion: (**A**) white blood cell count (WBC), (**B**) neutrophil percentage (Neu%), (**C**) eosinophil percentage (Eos%), (**D**) lymphocyte percentage (Lym%), (**E**) red blood cell count (RBC), (**F**) red cell distribution width-standard deviation (RDW-SD), (**G**) platelet count (PLT), and (**H**) plateletcrit (PCT%). *n* = 10 per group. (**I**–**L**) Plasma levels of pro-inflammatory cytokines: (**I**) tumor necrosis factor-α (TNF-α), (**J**) interleukin-1β (IL-1β), (**K**) interleukin-6 (IL-6), and (**L**) interleukin-17 (IL-17). *n* = 10 per group. (**M**–**P**) Myocardial tissue levels of pro-inflammatory cytokines: (**M**) TNF-α, (**N**) IL-1β, (**O**) IL-6, and (**P**) IL-17 (normalized to total protein). *n* = 8 per group. (**Q**) Representative immunohistochemical (IHC) staining of myeloperoxidase (MPO) in myocardium at the end of the 2 h immersion (scale bar: 20 μm; red arrow indicates MPO-positive neutrophils), *n* = 6 per group. * *p* < 0.05, ** *p* < 0.01, *** *p* < 0.001, and **** *p* < 0.0001; ns, not significant.

Such mitochondrial disruption releases damage associated molecular patterns (DAMPs) that trigger sterile inflammation [28]. We therefore examined hematological parameters and inflammatory mediators to assess the systemic and cardiac immune responses following cold seawater immersion. Compared with the Ctrl group, the SW group exhibited stress-induced leukocyte redistribution (decreased counts of white blood cel (WBC, *p* < 0.05, Figure 3A), reduced neutrophil (Neu%, *p* < 0.05, Figure 3B) and eosinophil (Eos%, *p* < 0.05, Figure 3C) proportions, and increased lymphocyte proportion (Lym%, *p* < 0.01, Figure 3D). Red blood cell count (RBC) remained unchanged (Figure 3E), while reduced red cell distribution width-standard deviation (RDW-SD) (*p* < 0.05, Figure 3F), indicating mild hematopoietic suppression. Platelet count and plateletcrit were markedly decreased (*p* < 0.001, *p* < 0.01, Figure 3G,H), suggesting consumptive thrombocytopenia.

In cardiac tissue, tumor necrosis factor-α (TNF-α) (*p* < 0.01, Figure 3M), IL-1β (*p* < 0.05, Figure 3N), and IL-6 (*p* < 0.01, Figure 3O) were significantly upregulated, indicating local myocardial inflammation. A decreasing trend in IL-17 expression was observed in the SW group (Figure 3P), suggesting early innate immunity activation without Th17 involvement. Consistent with local inflammation, plasma levels of IL-1β (*p* < 0.05, Figure 3J) and IL-6 (*p* < 0.0001, Figure 3K) were elevated, reflecting cytokine spillover from injured myocardium. In contrast, plasma TNF-α remained unchanged (ns, Figure 3I), likely due to its short half-life and the early detection window, while reduced IL-17 (*p* < 0.05, Figure 3L) confirmed the absence of systemic Th17 activation. Immunohistochemical staining for myeloperoxidase (MPO) revealed only scattered, focal positive signals in the myocardium of SW rats at this early time point (Figure 3Q), supporting incipient and low-grade neutrophil infiltration without extensive inflammatory cell recruitment. The dissociation between decreased circulating neutrophils and pro-inflammatory cytokines indicated that early myocardial inflammation was initiated primarily by stress-induced cellular damage and DAMP release, rather than massive myeloid cell infiltration. Collectively, these findings indicated that cold seawater immersion initiated local myocardial inflammation followed by systemic cytokine release, contributing to the initiation of cardiac injury at the early stage.

### 2.4. Transcriptomic Analysis Revealed a Pro-Inflammatory Signature in the Hypothermic Seawater-Exposed Heart

**Figure 4:**
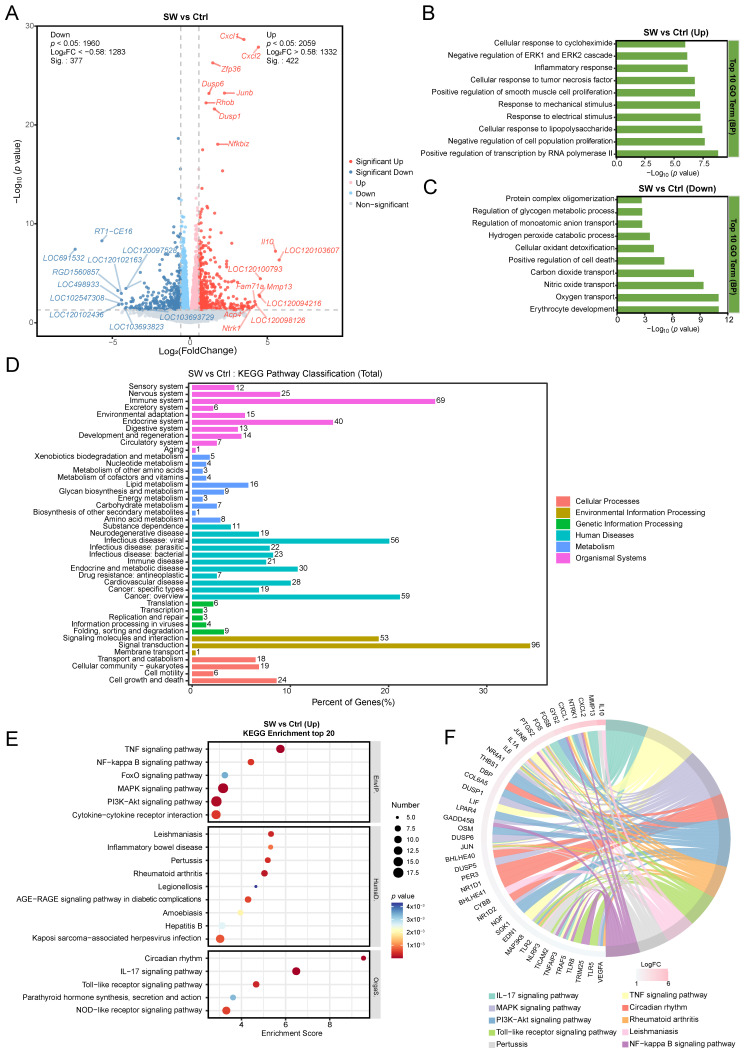
Transcriptomic profiling of cold seawater immersion-induced cardiac injury in rats. (**A**) Volcano plot illustrating differentially expressed genes (DEGs, *p* < 0.05, |log_2_FC| > 0.585) in cardiac tissue at the end of 2 h immersion from seawater-immersed (SW) versus control (Ctrl) rats. Red dots indicate upregulated genes, blue dots indicate downregulated genes, and gray dots indicate non-significant changes. (**B**,**C**) Gene Ontology (GO) enrichment analysis of DEGs: (**B**) Top biological processes enriched in upregulated genes, primarily involving inflammatory and stress responses; (**C**) Top biological processes enriched in downregulated genes, associated with metabolic homeostasis and gas exchange. (**D**) KEGG pathway classification histogram showing the distribution of DEGs across major functional categories. (**E**) Top 20 enriched KEGG pathways of upregulated genes, highlighting key inflammatory signaling pathways (e.g., TNF, NF-κB, MAPK, PI3K-Akt). (**F**) Circos plot visualizing the correlation between enriched KEGG pathways and corresponding differentially expressed genes, demonstrating coordinate upregulation of inflammatory modules (e.g., NF-κB, MAPK, *Nlrp3*) in the SW group. Data are presented based on RNA-sequencing results (*n* = 6 per group).

To characterize the transcriptional changes underlying cardiac injury induced by cold seawater immersion, we performed RNA-sequencing on cardiac tissues from Ctrl and SW rats. Volcano plot analysis identified 377 significantly downregulated and 422 significantly upregulated genes in the SW group compared with Ctrl (*p* < 0.05, |log_2_FC| > 0.58; Figure 4A). Prominently upregulated genes included pro-inflammatory chemokines (*Cxcl1*, *Cxcl2*), immediate early response genes (*Junb*, *Zfp36*), stress-responsive phosphatases (*Dusp1*, *Dusp6*), and inflammatory regulators (*Nfkbiz*, *Il10*). GO enrichment analysis of upregulated genes revealed significant enrichment in biological processes associated with inflammation and stress responses, including “inflammatory response,” “cellular response to tumor necrosis factor,” and “negative regulation of ERK1 and ERK2 cascade” (Figure 4B). In contrast, downregulated genes were enriched in processes related to metabolic homeostasis and gas exchange, such as “oxygen transport,” “nitric oxide transport,” “hydrogen peroxide catabolic process,” and “erythrocyte development” (Figure 4C). KEGG pathway classification showed that differentially expressed genes were mainly distributed across Organismal Systems (e.g., immune system, circulatory system) and Environmental Information Processing (e.g., signal transduction) (Figure 4D). KEGG enrichment analysis of upregulated genes identified activation of key inflammatory signaling pathways, including the TNF, NF-κB, mitogen-activated protein kinase (MAPK), and phosphatidylinositol 3-kinase-protein kinase B (PI3K-Akt) signaling pathways (Figure 4E). Circos plot visualization revealed coordinated transcriptional upregulation of inflammatory modules, including NF-κB signaling, MAPK signaling, and inflammasome components (*Nlrp3*), in the injured heart (Figure 4F). Collectively, these transcriptomic data reveal a distinct pro-inflammatory transcriptional signature in the SW group, providing a resource for further mechanistic investigations into the molecular pathways underlying hypothermic seawater-induced cardiac injury.

### 2.5. Cold Seawater Immersion Activated Cardiac Pattern Recognition Receptor (PRR) Signaling and the NF-κB/NLRP3/Pyroptosis Axis

**Figure 5:**
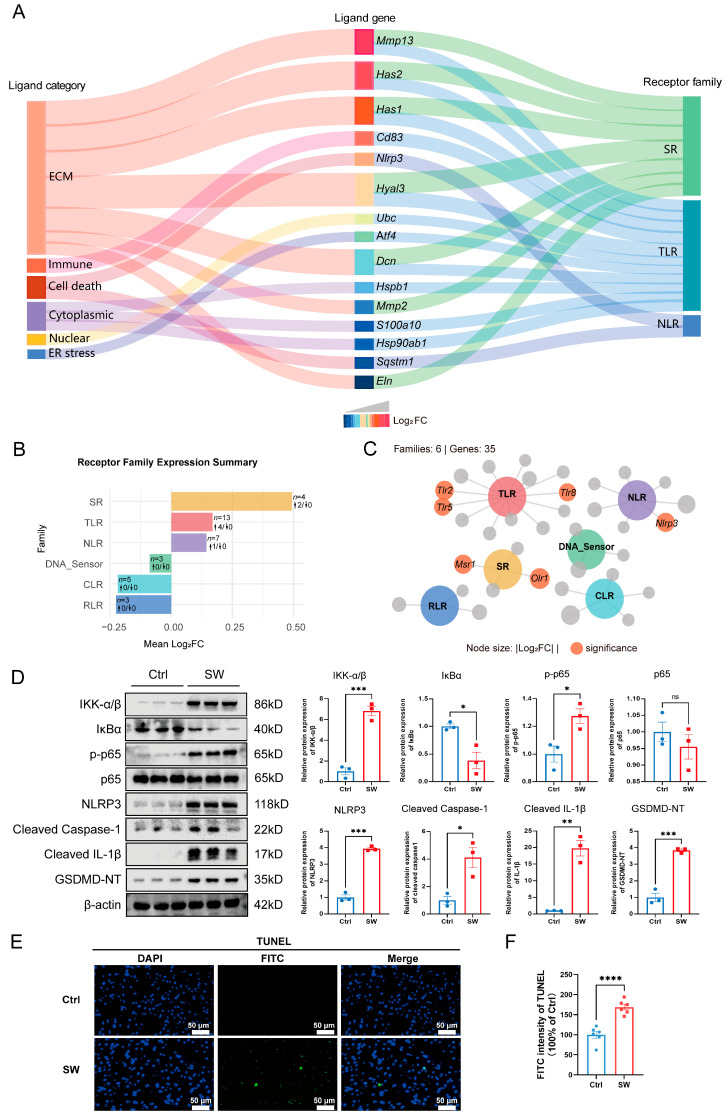
Cold seawater immersion activated pattern recognition receptor (PRR) signaling and the NF-κB/NLRP3/pyroptosis axis in rat myocardium. (**A**) Alluvial plot illustrating the interaction between differentially expressed ligand genes (categorized by biological function: ECM, immune, cell death, cytoplasmic, nuclear, ER stress) and three major PRR families (SR, TLR, NLR). Ligand color intensity reflects log_2_(FC) (blue to red = weak to strong) in the seawater-immersed (SW) versus control (Ctrl) group. (**B**) Bar chart summarizing mean log_2_(FC) of PRR family expression, showing prominent upregulation of the TLR family, followed by SR and NLR families. Upward arrows indicate significant upregulation, and downward arrows indicate significant downregulation. (**C**) Interaction network visualization of PRR signaling, highlighting upregulated TLR (*Tlr2*, *Tlr5*, *Tlr8*) and SR (*Msr1*, *Olr1*) genes, with *Nlrp3* in the NLR pathway (node size = |log_2_FC|, orange nodes = significant changes). (**D**) Western blot validation of the NF-κB/NLRP3/pyroptosis axis: representative blots and quantitative analysis of IKK-α/β, IκBα, phosphorylated p65 (p-p65), total p65, NLRP3, cleaved caspase-1, cleaved IL-1β, and GSDMD-NT (normalized to β-actin, *n* = 3 per group). (**E**,**F**) Representative images (**E**) and quantitative analysis (**F**) of TUNEL staining in rat myocardium at 2 h post immersion (scale bars = 50 μm). Data are presented as mean ± SEM (*n* = 6 per group); * *p* < 0.05, ** *p* < 0.01, *** *p* < 0.001, and **** *p* < 0.0001, ns, not significant.

To further dissect the inflammatory signaling networks underlying cardiac injury, we performed PRR transcriptomic analysis and validated key pathway activation at the protein level. Alluvial plot analysis revealed that differentially expressed ligand genes, categorized into extracellular matrix (ECM), immune, cell death, cytoplasmic, nuclear, and endoplasmic reticulum (ER) stress classes, were predicted to interact with three primary receptor families: scavenger receptors (SRs), Toll-like receptors (TLRs), and NOD-like receptors (NLRs) (Figure 5A). The top five potential damage-associated ligands were identified as *Mmp13*, *Has2*, *Has1*, *Cd83*, and *Nlrp3*, all of which were upregulated and mapped to the three receptor families. Analysis of receptor family expression demonstrated that the TLR family exhibited the most robust upregulation, followed by the SR family and NLR family, while other receptor families showed reduced expression (Figure 5B). Network visualization confirmed prominent upregulation of TLR cluster (*Tlr2*, *Tlr5*, *Tlr8*) and SR genes (*Msr1*, *Olr1*), with *Nlrp3* in the NLR signaling pathway (Figure 5C).

Given that TLR signaling converges on the NF-κB pathway, a master transcriptional regulator of pro-inflammatory cytokines, and that *Nlrp3* serves as the core component of the NLRP3 inflammasome responsible for pyroptotic cell death, we next validated this axis at the protein level. Western blot analysis confirmed activation of the NF-κB/NLRP3/pyroptosis axis (Figure 5D). Compared with the Ctrl group, SW exposure significantly increased the protein expression of IKK-α/β (*p* < 0.001), while markedly reducing IκBα levels (*p* < 0.05). Concomitantly, the phosphorylation level of p65 (p-p65) was significantly elevated (*p* < 0.05), whereas total p65 expression remained unchanged. These data confirm the activation of the canonical NF-κB signaling cascade in response to seawater immersion. Concurrently, downstream of NF-κB, SW treatment dramatically upregulated the expression of NLRP3 (*p* < 0.001), indicating enhanced transcriptional activation of the *Nlrp3* gene. Furthermore, the cleaved forms of caspase-1 (*p* < 0.001) and IL-1β (*p* < 0.01) were markedly increased in the SW group, demonstrating functional assembly and activation of the NLRP3 inflammasome. Finally, the N-terminal fragment of gasdermin D (GSDMD-NT), a hallmark of pyroptotic cell death, was significantly accumulated in SW-exposed myocardium (*p* < 0.001). Meanwhile, TUNEL staining showed that the SW group exhibited a marked increase in TUNEL-positive signals and extensive myocardial DNA damage (Figure 5E,F), providing morphological evidence for aggravated cardiomyocyte injury and further supporting the occurrence of NLRP3-mediated pyroptosis. Collectively, these findings validated the transcriptional PRR data at the protein level, confirming that cold seawater immersion triggered coordinated activation of the NF-κB pathway and the NLRP3/pyroptosis axis, which together orchestrated the inflammatory response and contributed to myocardial injury.

## 3. Discussion

Cold seawater immersion-induced cardiac arrest represents a life-threatening risk for maritime workers. Intervening before irreversible damage occurs is therefore essential for cardioprotection, and elucidating the early molecular mechanisms is of great clinical importance. In this study, we demonstrated that 2 h of immersion in 15 °C seawater induced cardiac dysfunction via the NF κB/NLRP3 pyroptosis signaling axis in rats. This pathway links seawater immersion hypothermic stress to inflammatory myocardial injury, and its identification opens possibilities for early therapeutic intervention.

Hypothermia induces a series of physiological changes. In a normal heart, mild hypothermia (e.g., 33 °C) reduces heart rate and increases myocardial contractility, while simultaneously impairing active ventricular relaxation [29]. Spontaneous bradycardia results in a prolonged diastole, which enhances ventricular filling and increases the preload. According to the Frank–Starling mechanism, this increase in initial myocardial fiber length leads to a more forceful contraction [30]. Mild therapeutic hypothermia thus potentiates myocardial contractility, which partially compensates for the concomitant diastolic dysfunction, thereby maintaining cardiac output. This hemodynamic compensatory mechanism constitutes a key theoretical underpinning for the clinical application of therapeutic hypothermia [31]. However, in our model of severe seawater immersion hypothermia, this compensatory mechanism was overwhelmed. Although the Frank Starling mechanism was activated to enhance myocardial contractility, cardiac output fell significantly, reflecting the compensatory limit of the heart under deep hypothermia. The marked decrease in cardiac output can result in inadequate tissue perfusion and compromised oxygen supply to multiple organs [32]. As a highly oxygen-dependent organ, the heart is particularly susceptible to oxygen insufficiency. Subsequent hypoperfusion impaired mitochondrial function, causing cellular energy depletion that inactivates energy-dependent ion transporters Na^+^/K^+^-ATPase and leads to cardiomyocyte swelling and rupture [33]. Consistently, we observed marked mitochondrial ultrastructural damage in the SW group. Moreover, the morphological pattern of mitochondria in SW group is consistent with recent findings showing that seawater immersion activates dynamin-related protein 1 and promotes its translocation from the cytoplasm to mitochondria in cardiomyocytes, leading to excessive mitochondrial fission and subsequent dysfunction [34]. Together, these observations suggest that mitochondrial dynamics represent a key mechanism linking cold seawater stress to myocardial injury and cardiac dysfunction.

Accidental hypothermia triggers a complex and biphasic immune response. While mild hypothermia (32 °C) has been shown to directly suppress macrophage immune activation in vitro [35], the inflammatory landscape shifts dramatically when hypothermia induces cellular damage. Cellular injury from severe hypothermia releases DAMPs that activate innate immunity and lead to systemic inflammatory response syndrome [36]. Mitochondrial damage serves as a critical hub linking hypothermia and inflammatory activation. When core temperature falls below 32 °C or hypothermia persists excessively, mitochondrial homeostasis collapses, promoting the extracellular release of DAMPs such as mitochondrial DNA (mtDNA) and reactive oxygen species (ROS) [37,38]. These DAMPs are recognized by PRRs on the surface of cardiomyocytes and adjacent immune cells, thereby initiating local inflammatory responses. The present study demonstrated that inflammation induced by cold seawater immersion exhibited remarkable spatiotemporal restriction. Protein levels of TNF-α, IL-1β, and IL-6 were significantly upregulated in cardiac tissue, indicating activation of innate immunity in the myocardium. In contrast, TNF-α was not significantly elevated in plasma, which may reflect either a true delay in systemic release or lower assay sensitivity for circulating TNF-α at this early time point. This pattern resembles early acute myocardial infarction, where local cytokine activation precedes systemic inflammation [39]. Consistent with local activation, the genes coding neutrophil chemokines (*Cxcl1*, *Cxcl2*) were upregulated, accompanied by a reduced percentage of circulating neutrophils; however, MPO immunohistochemistry at this 2 h time point showed only scattered MPO-positive cells, indicating that while chemotactic signals have been initiated, significant neutrophil infiltration had not yet occurred at this early stage. Thus, the 2 h time point captures an early, localized inflammatory phase driven by DAMPs, likely derived from mitochondria.

PRRs are core sensors of the innate immune system that recognize pathogen associated molecular patterns (PAMPs) and DAMPs. In addition to canonical PRR families including TLRs, NLRs, retinoic acid-inducible gene I-like receptors, C-type lectin receptors, and absent in melanoma 2-like receptors, SRs act as nonclassical PRRs and play important roles in sterile inflammation [40,41]. Our transcriptomic analysis revealed coordinated activation of SRs, TLRs, and NLRs in cold seawater immersion-induced myocardial injury, suggesting a multi-tiered sensing network rather than reliance on a single receptor family. TLRs, as primary DAMP sensors in extracellular and endosomal compartments [42], were broadly upregulated, indicating that TLR-mediated damage recognition is a dominant pathway driving myocardial inflammation. Among the DAMPs released from cold-injured cardiomyocytes, cardiolipin, membrane phospholipids, and extracellular matrix degradation products may activate the NF-κB pathway via a myeloid differentiation primary response-88 dependent mechanism, initiating inflammation and providing the priming signal for inflammasome assembly [36]. NLRP3, a cytosolic NLR essential for sensing mitochondria DAMPs [43], was upregulated at both mRNA and protein levels, acting as a key amplifier of inflammation. NLRP3 inflammasome activation relies on two-signal mechanism: the priming signal mediated by TLR/NF-κB signaling upregulates *Nlrp3* and *Il1b* transcription; the activation signal triggered by mtROS, mtDNA, and other mitochondrial DAMPs promotes inflammasome assembly and caspase 1 activation, leading to the maturation and release of IL-1β/IL-18 [43]. Notably, SR family members were also transcriptionally upregulated. SRs are mainly expressed on myeloid cells such as macrophages and recognize a broad range of endogenous and exogenous ligands [44]. Classic SRs such as MSR1 do not independently initiate inflammatory responses, but can efficiently internalize ligands including oxidized lipids and damaged mitochondrial debris and deliver them to intracellular compartments harboring TLRs and NLRs, thereby enhancing ligand accessibility and amplifying downstream signaling [45]. Thus, we propose that in cold seawater immersion-induced myocardial injury, SRs mediate the recognition and internalization of damage associated ligands, TLRs sense DAMPs at the membrane and endosomal compartments and drive NF-κB dependent proinflammatory transcription, and NLRs detect cytosolic DAMPs to trigger inflammasome activation. This coordinated SR-TLR-NLR sensing network has not been previously described in hypothermic cardiac injury and may represent a specialized response to the complex DAMP milieu generated by cold seawater stress. These findings are based on transcriptomic and protein evidence, and further functional studies are required to verify the precise molecular interactions and functional dependencies.

The ultimate outcome of inflammasome activation is pyroptosis, a form of inflammatory cell death that serves as a critical link between inflammatory signaling pathways and cardiac dysfunction [46]. Pyroptosis is mediated by the gasdermin family of proteins, with GSDMD being the key executor downstream of caspase-1 [47]. Activated caspase-1 cleaves GSDMD to release its N-terminal effector domain, which oligomerizes on the cell membrane to form pores, disrupts membrane integrity, and causes cell swelling, lysis, and the release of intracellular contents. Meanwhile, caspase-1 cleaves pro-IL-1β and pro-IL-18 to generate mature IL-1β and IL-18, which are secreted extracellularly through GSDMD pores to further amplify the inflammatory response [47]. Notably, a bidirectional amplifying cycle exists between pyroptosis and mitochondrial damage. On the one hand, mitochondrial damage-associated molecular patterns (mtROS, mtDNA, ATP, etc.) act as key activating signals for the NLRP3 inflammasome [48,49,50]. On the other hand, IL-1β released during pyroptosis further impairs mitochondrial function in neighboring cells [51]. This mechanism explains why marked mitochondrial structural damage and inflammatory activation are already observable as early as 2 h after immersion in our model. IL-1β has been shown to directly impair cardiac diastolic function by increasing myocardial mitochondrial ROS production, inducing cardiomyocyte hypertrophy, promoting extracellular matrix remodeling, and reducing myocardial compliance [52]. This suggests that the impaired diastolic relaxation or reduced myocardial compliance induced by cold seawater immersion is closely associated with IL-1β released via pyroptotic pores. Furthermore, the ionic imbalance caused by GSDMD pore formation disturbs cardiomyocyte action potentials and increases susceptibility to arrhythmias [53,54]. On the basis of bradycardia already induced by cold seawater immersion, electrophysiological disorders and myocardial dysfunction caused by pyroptotic pores further exacerbate hemodynamic deterioration. Collectively, the present study demonstrates for the first time that cold seawater immersion triggers the NLRP3/caspase 1/GSDMD pyroptotic pathway, mediating inflammatory cardiomyocyte death and contributing to cardiac dysfunction. The early activation of this positive feedback loop highlights a potential therapeutic window for interventions targeting pyroptosis before irreversible damage occurs.

**Figure 6:**
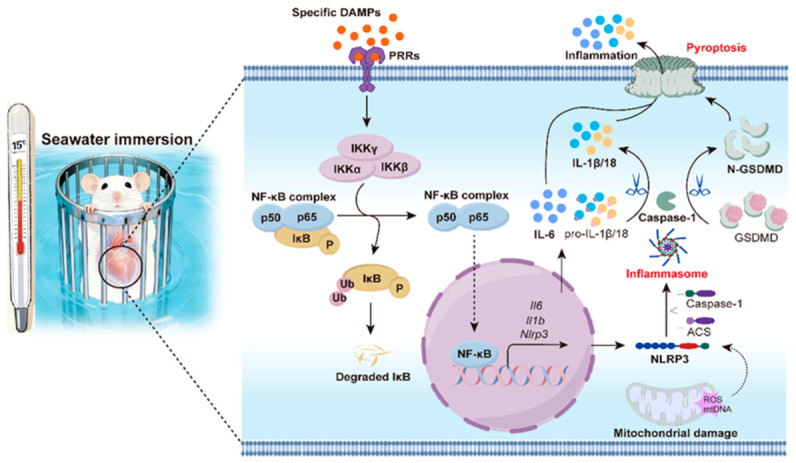
Schematic model of cold seawater immersion-induced myocardial injury in rats. Hypothermic (15 °C) seawater immersion triggered cardiomyocyte damage and the release of damage-associated molecular patterns (DAMPs). DAMPs were sensed by a coordinated network of SRs, TLRs, and NLRs, leading to the activation of downstream signaling cascades, including the IKK complex. This led to phosphorylation and degradation of IκBα, enabling nuclear translocation of NF-κB (p65). Nuclear NF-κB drove transcription of *Nlrp3* and pro-inflammatory cytokines, priming the NLRP3 inflammasome. Activated caspase-1 executed pyroptosis via GSDMD cleavage and releases mature IL-1β, driving myocardial inflammation and dysfunction.

In summary, we established a core mechanistic model of cold seawater immersion-induced myocardial injury. The cold seawater immersion stress disrupts myocardial mitochondrial structure, leading to DAMP release. These DAMPs (e.g., mtDNA, ROS) may be coordinately recognized by the SR–TLR–NLR network, initiating NF-κB signaling and NLRP3 inflammasome assembly, ultimately triggering GSDMD-mediated pyroptosis and cardiac dysfunction (Figure 6). This study addresses several knowledge gaps in cold seawater-induced cardiac injury. We identified a unique phenotype of Frank Starling compensatory enhancement in the context of bradycardia, clarifying the key transition from compensation to decompensation. Additionally, we demonstrated that mitochondrial ultrastructural damage precedes histological alterations, shifting the potential intervention window to the stage of mitochondrial dysfunction. Unlike prior studies focusing on individual PRRs, we systematically characterized the coordinated SR–TLR–NLRs sensing network and validated the NF-κB/NLRP3/pyroptosis cascade. Furthermore, we linked pyroptosis to diastolic dysfunction, offering a novel pathophysiological framework for cold-related myocardial injury.

This study has several limitations. The single time point design prevents definitive clarification of the temporal sequence between mitochondrial damage and inflammatory activation. Observational data lack interventional evidence to confirm causal relationships within the pathway. Tissue homogenate analysis cannot distinguish the contributions of cardiomyocytes and immune cells to pyroptosis. Moreover, the exclusive use of male rats introduces species and sex biases, limiting clinical extrapolation. Future research will adopt a multi-time point design to identify critical intervention windows, use specific inhibitors to verify the causal role of the NF-κB/NLRP3/pyroptosis axis, and apply single-cell technologies to define the core effector cells of pyroptosis. Cross-species validation and evaluation of clinically approved drugs will also be conducted to accelerate the translational application of these findings.

## 4. Materials and Methods

### 4.1. Animals and Seawater Immersion Model

Male Sprague-Dawley rats (7-week-old, weighing 220 ± 10 g) were purchased from Shanghai Regen Biotechnology Co., Ltd. (Shanghai, China). All rats were housed in the specific pathogen-free barrier environment of the Animal Experiment Center of Naval Medical University. Rats were housed 5 per cage, individually numbered via ear tags, and acclimatized for 1 week prior to experimentation. All animal procedures were approved by the Shanghai Changhai Hospital Ethics Committee (No. 2020-0307), and conformed to the NIH Guide for the Care and Use of Laboratory Animals. Rats were randomly divided into a blank control group and a seawater immersion hypothermia model group (random number table method). A dedicated experimental platform for seawater immersion hypothermia in rats (Patent No.: CN218606938U) was prepared one night before the experiment. Artificial seawater was prepared by dissolving 30 g of artificial sea salt (Guangzhou Yi’er Bioengineering Co., Ltd., Guangzhou, China) in 1000 mL of water, and pre-cooled to 15 °C for standby. On the day of experimentation, rats in the model group were immersed in pre-cooled seawater (15 °C) under conscious status to induce hypothermia. For continuous ECG monitoring, rats were immersed for up to 360 min to observe dynamic heart rate changes; for all other experiments, rats were immersed for 2 h and euthanized at the end of the 2 h immersion to assess early pathological and molecular changes. Rats in the control group were raised routinely without any intervention. The sample size for each experiment is specified in the corresponding subsection and figure legend.

### 4.2. ECG Data Acquisition and Analysis

ECG signals were collected and analyzed using an 8-channel clip-on electrocardiograph (provided by Prof. Chuantao Li’s research group, Naval Special Medical Center, Shanghai, China). Before experimentation, rats (*n* = 8 per group) were fixed in a cage-type holder and placed steadily on the seawater immersion platform. One end of the lead wire was connected to the 8-channel host, and the other end was clipped to the bilateral forepaws of rats via metal ear clips to stably guide ECG signals. ECG signals were transmitted to a supporting computer in real-time via a Wi-Fi module. The Wireless Acquisition software (version 1.0.0.0) was used to record continuous ECG within 0–360 min after seawater immersion, with parameters set as follows: sampling rate of 250 Hz, amplitude range of ±300 μV, and band-pass filter of 1–45 Hz. After exporting raw ECG data, waveform analysis and extraction of heart rate and other core ECG parameters were performed using MATLAB R2024a (MathWorks, Natick, MA, USA) and LabChart 8 (ADInstruments, Sydney, NSW, Australia).

### 4.3. Echocardiography

Chest hair of rats (*n* = 6 per group) was removed one day before the experiment to reduce ultrasound interference. On the day of examination, rats were anesthetized with isoflurane (RWD Life Science, Shenzhen, China) inhalation, fixed steadily on a detection plate, and coated with medical ultrasound coupling agent (Hainuo, Xiangtan, Hunan, China) on the chest. Cardiac ultrasound was performed using an HM70 EVO ultrasound diagnostic system (Samsung, Seoul, Republic of Korea). A 22 MHz linear array probe was used to collect long-axis views of the heart via transthoracic approach; left ventricular wall thickness, cavity diameter and ejection fraction were measured via M-mode ultrasound. A 9 MHz phased array probe was used to collect apical four-chamber views, and mitral inflow velocity spectrum (E and A peaks) was measured via pulsed-wave (PW) Doppler to calculate the E/A ratio for evaluating cardiac diastolic function.

### 4.4. Blood Sample Analysis

After 2 h immersion, rats (*n* = 10 per group) were anesthetized with 2% pentobarbital sodium (Sigma-Aldrich, St. Louis, MO, USA), and blood was collected from the abdominal aorta into vacuum blood collection tubes containing EDTA anticoagulant. A total of 100 μL whole blood was used for blood routine detection via an automatic hematology analyzer (BC-5100, Mindray Biomedical Electronics Co., Ltd., Shenzhen, China). The remaining whole blood was allowed to stand at room temperature for 2 h, then centrifuged at 3000× *g* rpm for 15 min at 4 °C to isolate plasma supernatant. Plasma biochemical indicators including LDH, AST, α-HBDH, CK-MB and CK were detected using an automatic biochemical analyzer (BK-28, Shandong Baoke Biotechnology Co., Ltd., Jinan, Shandong, China).

### 4.5. Enzyme-Linked Immunosorbent Assay (ELISA)

Plasma levels (*n* = 10 per group) of cTnT, cTnI, BNP, TNF-α, IL-1β, IL-6 and IL-17 were measured using rat-specific ELISA kits (Jiangsu Meimian industrial Co., Ltd., Yancheng, Jiangsu, China) following the manufacturer’s instructions. Heart tissues (*n* = 8 per group) were homogenized and centrifuged to collect supernatant, and inflammatory indicators (TNF-α, IL-1β, IL-6, IL-17) were detected using the same ELISA kits. The absorbance of all ELISA samples was measured using a microplate reader (Labsystems Multiskan MS, Vantaa, Finland).

### 4.6. Transmission Electron Microscopy (TEM)

Rats were deeply anesthetized with 2% pentobarbital sodium (Sigma-Aldrich, St. Louis, MO, USA), and left ventricular myocardial tissues (*n* = 3 per group) were quickly excised and immediately placed in a Petri dish containing pre-cooled electron microscope fixative (Servicebio, Wuhan, China). Tissues were precisely cut into 1 mm^3^ blocks and transferred to fresh fixative for static fixation at 4 °C. Subsequent sample embedding, sectioning and TEM imaging were entrusted to Servicebio (Wuhan, China). In brief, samples were post-fixed with 1% osmium acid (Ted Pella Inc., Redding, CA, USA) and dehydrated in a graded ethanol series. Infiltration and embedding were conducted with a mixture of acetone and SPI-Pon 812 (SPI, West Chester, PA, USA), followed by drying and curing. Resin blocks were cut into 1.5 μm semi-thin sections with a semi-thin microtome (Daitome Ultra 45°, Quakertown, PA, USA) and further processed into 60–80 nm ultra-thin sections with an ultra-microtome (Leica UC7, Leica, Wetzlar, Germany). Sections were mounted onto 150-mesh formvar-coated copper grids. Staining was performed in sequence with 2% uranyl acetate (SPI, West Chester, PA, USA) saturated alcohol solution in the dark and 2.6% lead citrate (Sigma, Burlington, MA, USA) solution protected from carbon dioxide. Myocardial ultrastructure was observed and images were captured using a transmission electron microscope (Hitachi HT7800, Tokyo, Japan).

### 4.7. Cardiac Transcriptome Sequencing and Analysis

Rats (*n* = 6 per group) were anesthetized with 2% pentobarbital sodium (Sigma-Aldrich, St. Louis, MO, USA), and heart tissues were quickly harvested and immediately frozen at −80 °C for standby. Total RNA was extracted from heart tissues using the TRIzol method, and RNA purity and integrity were verified by agarose gel electrophoresis and NanoDrop spectrophotometer (Thermo Scientific, Wilmington, DE, USA); qualified samples were stored at −80 °C. The RNA libraries were constructed and sequenced by OE Biotech Co., Ltd. (Shanghai, China). A cDNA library was constructed using the Illumina TruSeq Stranded mRNA Library Prep Kit (Illumina, San Diego, CA, USA), library quality was assessed using an Agilent 2100 Bioanalyzer (Agilent Technologies, Santa Clara, CA, USA), and sequencing was performed on the Illumina NovaSeq 6000 platform (Illumina, San Diego, CA, USA). Raw sequencing data were quality-controlled by FastQC (version 0.7.2) and filtered by Trimmomatic (version 0.39) to remove low-quality sequences, yielding high-quality clean reads. Reads were assembled using Trinity (version 2.15.2) and mapped to the rat reference genome. Unigene expression was quantified and converted to fragments per kilobase million (FPKM) values. Differentially expressed genes (DEGs) were screened using DESeq2 (version 1.22.2) with thresholds of *p* < 0.05 and |log_2_(Fold Change)| ≥ 0.585. GO functional annotation and KEGG pathway enrichment analysis were performed using the clusterProfiler package in R to elucidate core biological functions and signaling pathways. Bioinformatic visualization was performed using the OECloud tools at https://cloud.oebiotech.com/task/ (accessed on 10 October 2025).

### 4.8. Western Blot Analysis

Heart tissues (*n* = 3 per group) were homogenized on ice with RIPA lysis buffer (Beyotime, Shanghai, China). Centrifugation was conducted at 14,000× *g* rpm for 15 min at 4 °C to collect the supernatant. Protein concentration was determined with a BCA protein assay kit (Thermo Scientific, Waltham, MA, USA). Protein concentration was adjusted to 2 μg/μL, and protein denaturation was achieved by boiling in a water bath for 10 min to prepare loading samples. SDS-PAGE was performed with prefabricated 10% separating gel and stacking gel (Yamei Biotechnology Co., Ltd., Shanghai, China). Protein sample (20 μg per well) and pre-stained protein Marker (Thermo Scientific, Waltham, MA, USA) were loaded into each well. Electrophoresis was initiated at 80 V and adjusted to 120 V until the separation process was finished. Membrane transfer was carried out after PVDF membranes (0.45 μm, Millipore, Billerica, MA, USA) were activated with methanol. Proteins were transferred onto PVDF membranes via wet blotting at 200 mA for 2 h. Membrane blocking was performed with 5% bovine serum BSA (Sigma, Burlington, MA, USA) prepared in TBST buffer (Servicebio, Wuhan, China), and incubation was conducted on a shaker for 2 h at room temperature. Primary antibody incubation was performed with diluted primary antibodies overnight for 14 h at 4 °C. Membranes were washed with TBST three times for 10 min each after incubation. The following primary antibodies were used at the indicated dilutions: NLRP3 (PT0049R, Immunoway, San Jose, CA, USA, 1:1000), Cleaved-Caspase-1 (Asp297) (#4199T, Cell Signaling Technology, Danvers, MA, USA, 1:1000), Cleaved-IL-1β (Asp117) (#63124, Cell Signaling Technology, Danvers, MA, USA, 1:1000), Cleaved N-terminal GSDMD (PT0680R, Immunoway, San Jose, CA, USA, 1:2000), IKK-α/β (PT0435R, Immunoway, San Jose, CA, USA, 1:1000), IκBα (#4812S, Cell Signaling Technology, Danvers, MA, USA, 1:1000), NF-κB p65 (D14E12), (#8242T, Cell Signaling Technology, Danvers, MA, USA, 1:1000), Phospho-NF-κB p65 (Ser536, 93H1) (#3033S, Cell Signaling Technology, Danvers, MA, USA, 1:1000), BiP (#3183, Cell Signaling Technology, Danvers, MA, USA, 1:1000), GPX-4 (sc-166120, Santa Cruz Biotechnology, Dallas, TX, USA, 1:500), and beta-Actin (8H10D10) (#3700, Cell Signaling Technology, Danvers, MA, USA, 1:1000). Secondary antibody incubation was performed with goat anti-rabbit IgG secondary antibody (#7074P2, Cell Signaling Technology, Danvers, MA, USA) for rabbit primary antibodies, and anti-mouse IgG HRP-linked antibody (#7076, Cell Signaling Technology, Danvers, MA, USA) for mouse primary antibody, at a dilution of 1:2000 for 1 h at room temperature. Membranes were washed with TBST three times for 10 min each after incubation. Enhanced chemiluminescence reagent was added at a 1:1 dilution (Millipore, Billerica, MA, USA), and imaging was developed with the Bio-Rad ChemiDoc imaging system (Bio-Rad, Hercules, CA, USA). Three biological replicates were set for each group, and quantitative gray value analysis of protein bands was conducted with ImageJ software (version 1.48q).

### 4.9. Tissue ATP Content Detection

Tissue ATP levels were detected using an enhanced ATP assay kit (Beyotime Biotechnology, Shanghai, China). Tissue samples (*n* = 10 per group, 20 mg per sample) were homogenized in 200 μL lysis buffer and centrifuged at 12,000× *g* for 5 min at 4 °C using centrifuge (MicroCL 17R, Thermo Fisher Scientific, Waltham, MA, USA) to collect supernatants. ATP working solution and serially diluted ATP standards were freshly prepared on ice. Each well was pre-incubated with 100 μL working solution for 3 min to eliminate background signals, followed by the addition of samples or standards. Luminescence intensity was measured immediately using Multimode Microplate Reader (Varioskan Flash, Thermo Fisher Scientific, Waltham, MA, USA), and ATP concentrations were calculated from the standard curve. Results were normalized to total protein content and expressed as nmol/mg protein.

### 4.10. Tissue MDA Content Detection

Tissue MDA levels (*n* = 10 per group) were determined using a lipid oxidation assay kit (Beyotime Biotechnology, Shanghai, China). MDA standards were serially diluted with ultrapure water (Aiken Water Elf, Hangzhou, China) to establish a standard curve. PBS blank, standards, and tissue supernatants (0.1 mL each) were mixed with 0.2 mL MDA working solution, boiled for 15 min, cooled, and centrifuged at 3000× *g* for 10 min. The supernatant absorbance was detected at 532 nm using Multimode Microplate Reader (Varioskan Flash, Thermo Fisher Scientific, Waltham, MA, USA). MDA contents were calibrated by the standard curve and normalized to total protein, presented as μmol/mg protein.

### 4.11. Tissue Paraffin Embedding

Rat heart tissues (*n* = 6 per group) were fixed in 4% paraformaldehyde solution (Servicebio, Wuhan, China) for 12–24 h. After trimming, qualified tissue samples were placed in labeled dehydration cassettes and subjected to gradient dehydration and wax infiltration using a tissue dehydrator (Servicebio, Wuhan, China). The procedure included 75% ethanol (1.5 h), 85% ethanol (1.5 h), 90% ethanol (1.5 h), 95% ethanol (1–1.5 h), absolute ethanol I/II (1–1.5 h each), alcohol-xylene (5–10 min), xylene I/II (10–20 min each), and 65 °C molten paraffin I/II/III (0.5 h, 1 h, 1.5 h). Subsequently, tissues were embedded in paraffin using an embedding machine (Servicebio, Wuhan, China). The embedded blocks were cooled on a freezing table (Servicebio, Wuhan, China) at −20 °C, trimmed after complete solidification, and stored for subsequent staining.

### 4.12. Hematoxylin and Eosin (H&E) Staining

Paraffin sections (*n* = 6 per group) were dewaxed and rehydrated using xylene and gradient ethanol. Sections were stained with hematoxylin (Servicebio, Wuhan, China) for 3 min, rinsed with running water, differentiated in hematoxylin differentiation solution (Servicebio, Wuhan, China), and blued in bluing solution. Subsequently, sections were stained with eosin solution (Servicebio, Wuhan, China) for 1–2 min, followed by routine gradient dehydration, transparency with xylene, and neutral balsam mounting. Tissue morphological changes were observed and photographed under a light microscope (SWe-CX63, Servicebio, Wuhan, China).

### 4.13. Immunohistochemical Staining

Paraffin sections (*n* = 6 per group) were routinely dewaxed and rehydrated with xylene and gradient ethanol. Antigen retrieval was performed in citrate buffer (Servicebio, Wuhan, China) using a pressure cooker for 2 min. After PBS washing (3 × 5 min), sections were blocked with 3% methanol-hydrogen peroxide and 3% BSA to eliminate endogenous peroxidase activity and non-specific binding. Sections were incubated with myeloperoxidase primary antibody (Servicebio, Wuhan, China) at a 1:1000 dilution overnight at 4 °C, followed by incubation with HRP-conjugated secondary antibody (GB23301, 1:200, Servicebio, Wuhan, China) at room temperature for 1 h. Positive immunoreactivity was visualized via DAB chromogen staining (Servicebio, Wuhan, China). Nuclei were counterstained with hematoxylin. Finally, sections were dehydrated, cleared, and mounted for observation. All sections were scanned with a digital slice scanner (LG-S80, Servicebio, Wuhan, China).

### 4.14. TUNEL Fluorescence Staining

Paraffin sections (*n* = 6 per group) were dewaxed and rehydrated routinely. Tissues were treated with diluted Proteinase K (Servicebio, Wuhan, China) working solution (1:9 in PBS) at 37 °C for 20 min, permeabilized at room temperature for 20 min, and equilibrated with TUNEL buffer for 10 min. The TUNEL reaction mixture (TDT enzyme:dUTP:buffer = 2:5:50, Servicebio, Wuhan, China) was freshly prepared and applied to tissues, followed by incubation in a humidified chamber at 37 °C for 1 h. After PBS washing, nuclei were counterstained with DAPI (Servicebio, Wuhan, China) in the dark for 10 min. Sections were mounted with anti-fluorescence quenching medium (Servicebio, Wuhan, China). Fluorescence signals were observed and imaged under a fluorescence microscope (Eclipse C1, Nikon, Tokyo, Japan) using DAPI and FITC channels with corresponding excitation and emission wavelengths.

### 4.15. Statistical Analysis

All statistical analyses were performed using GraphPad Prism 9.0 and R software (version 4.2.1). Data are presented as the mean ± SEM or box-and-whisker plots (median with interquartile range, minimum to maximum) with individual data points overlaid, as indicated in the corresponding figure legends. For comparisons between two groups, unpaired two-tailed Student’s *t*-test was used. For time-course dynamic data, repeated-measures ANOVA followed by post hoc tests was conducted to assess inter-group differences at each time point. *n* represents the number of biological replicates. Statistical significance was set at *p* < 0.05. All experiments were performed in at least triplicate to ensure reproducibility.

## 5. Conclusions

This study confirms that 15 °C seawater immersion stress disrupts myocardial mitochondrial homeostasis, triggering the sequential activation of the NF-κB/NLRP3/caspase 1/GSDMD pyroptosis pathway, which ultimately mediates inflammatory cardiomyocyte death and cardiac dysfunction. Integrating transcriptomic and protein validation data, we defined the core pathological cascade linking mitochondrial damage to DAMP release, subsequent inflammasome activation, and ultimately pyroptotic cell death under cold seawater stress and elucidated the pivotal role of pyroptosis in cardiac decompensation. These findings fill the mechanistic gap in cold seawater-induced myocardial injury and provide a solid experimental basis and novel insights for developing pyroptosis-targeted therapies and optimizing rescue strategies for cold stress cardiac injury in maritime and military medicine.

## Data Availability

The original contributions presented in this study are included in the article. Further inquiries can be directed to the corresponding authors.

## Abbreviations

The following abbreviations are used in this manuscript:

α-HBDH	α-hydroxybutyrate dehydrogenase
AST	aspartate transaminase
BNP	B-type natriuretic peptide
CK	creatine kinase
CK-MB	creatine kinase-MB
CO	cardiac output
cTnI	cardiac troponin I
cTnT	cardiac troponin T
DAMPs	damage-associated molecular patterns
ECM	extracellular matrix
Eos%	eosinophil percentage
ER	endoplasmic reticulum
GSDMD	gasdermin D
GSDMD-NT	GSDMD N-terminal fragment
HPA	hypothalamic–pituitary–adrenal
LDH	lactate dehydrogenase
LVEDD	left ventricular end-diastolic diameter
LVESD	left ventricular end-systolic diameter
LVEF	left ventricular ejection fraction
LVFS	left ventricular fractional shortening
Lym%	lymphocyte percentage
MAPK	mitogen-activated protein kinase
mtDNA	mitochondrial DNA
Neu%	neutrophil percentage
NF-κB	nuclear factor-kappaB
NLR	NOD-like receptor
NLRP3	NLR family pyrin domain containing 3
PAMPs	pathogen-associated molecular patterns
PCT	plateletcrit
PI3K-Akt	phosphatidylinositol 3-kinase-protein kinase B
PLT	platelet
PRR	pattern recognition receptor
RBC	red blood cell
RDW-SD	red cell distribution width-standard deviation
ROS	reactive oxygen species
SR	scavenger receptor
SV	stroke volume
SW	seawater immersion
TEM	transmission electron microscopy
TLR	Toll-like receptor
TNF-α	tumor necrosis factor-α
WBC	white blood cell

## References

[1] International Institute of Marine Surveying (IIMS) Annual Overview of Marine Casualties and Incidents 2025 Report Published by EMSA. https://www.iims.org.uk/annual-overview-of-marine-casualties-and-incidents-2025-report-published-by-emsa/.

[2] Marine Accident Investigation Branch Planning and Preparation Vital to Reduce Man Overboard Fatalities. https://www.gov.uk/government/news/planning-and-preparation-vital-to-reduce-man-overboard-fatalities.

[3] National Center for Cold Water Safety Why Cold Water Is Dangerous. https://www.coldwatersafety.org/the-danger.

[4] Peter S., Michaelis A., Wagner R., Marshall R.P., Bovet M., Weickmann J., Weidenbach M., Dähnert I., Paech C. (2026). Cold shock response in healthy children: Reassessment and first comparison between cold and warm water immersion. Front. Sports Act. Living.

[5] Jones D.M., Weller R.S., McClintock R.J., Roberts N., Zheng W., Dunn T.L. (2023). Prevalence of hypothermia and critical hand temperatures during military cold water immersion training. Int. J. Circumpolar Health.

[6] Lucas D.L., Case S.L., Lincoln J.M., Watson J.R. (2017). Factors associated with crewmember survival of commercial fishing vessel sinkings in Alaska. Saf. Sci..

[7] Benham D.A., Vasquez M.C., Kerns J., Checchi K.D., Mullinax R., Edson T.D., Tadlock M.D. (2023). Injury trends aboard US Navy vessels: A 50-year analysis of mishaps at sea. J. Trauma. Acute Care Surg..

[8] Masoumi Shahrbabak S., Bouzid Z., Inan O.T., Hahn J.O. (2025). Physiology and enabling technologies for quantitative assessment of survivability during cold water immersion and rewarming: A review. Prog. Biomed. Eng..

[9] Walpoth B.H., Maeder M.B., Courvoisier D.S., Meyer M., Cools E., Darocha T., Blancher M., Champly F., Mantovani L., Lovis C. (2021). Hypothermic Cardiac Arrest—Retrospective cohort study from the International Hypothermia Registry. Resuscitation.

[10] Hanneman K., Alahmad B., Ghosh A., Khatana S.A.M., Huang M., Liu J., Abadi A., Khraishah H., Beckie T., Rajagopalan S. (2026). Nonoptimal Temperature and Cardiovascular Health: A Scientific Statement From the American Heart Association. Circulation.

[11] Dietrichs E.S., McGlynn K., Allan A., Connolly A., Bishop M., Burton F., Kettlewell S., Myles R., Tveita T., Smith G.L. (2020). Moderate but not severe hypothermia causes pro-arrhythmic changes in cardiac electrophysiology. Cardiovasc. Res..

[12] Bjertnæs L.J., Næsheim T.O., Reierth E., Suborov E.V., Kirov M.Y., Lebedinskii K.M., Tveita T. (2022). Physiological Changes in Subjects Exposed to Accidental Hypothermia: An Update. Front. Med..

[13] Varon J. (2025). Hypoxemia in hypothermic cardiac arrest: Why one number should not decide a life. Resuscitation.

[14] Mendrala K., Darocha T., Podsiadło P., Hymczak H., Witt-Majchrzak A., Nowak E., Pluta M., Barteczko-Grajek B., Dąbrowski W., Kosiński S. (2025). Oxygenation metrics have limited prognostic value in non-asphyxial hypothermic cardiac arrest. Resuscitation.

[15] Pei Z., Xiong Y., Jiang S., Guo R., Jin W., Tao J., Zhang Z., Zhang Y., Zou Y., Gong Y. (2024). Heavy Metal Scavenger Metallothionein Rescues Against Cold Stress-Evoked Myocardial Contractile Anomalies Through Regulation of Mitophagy. Cardiovasc. Toxicol..

[16] Zuo S., Fu W., Ma W., Wang H., Feng S., Sun H., Zhang Z., Li L., Xue Y., Xu H. (2026). Ginsenoside Rg2 ameliorates acute cold exposure/rewarming-induced myocardial injury via modulating HMGB1/TLR4/NF-κB and PGC-1α signaling pathways: Role of SIRT1. Phytomedicine.

[17] Kong X., Liu H., He X., Sun Y., Ge W. (2020). Unraveling the Mystery of Cold Stress-Induced Myocardial Injury. Front. Physiol..

[18] Junaid M., Mahmud-Or-Rashid M. (2024). Computational insights into survival durations and prehospital interventions in accidental cold-water immersion: A comprehensive analysis of fresh and saltwater temperatures. Heliyon.

[19] Xing L., Li H., Miao D., Wei H., Zhang S., Xue Q., Wang H., Li J. (2024). Intermittent and mild cold stimulation enhances immune function of broilers via co-regulation of CIRP and TRPM8 on NF-κB and MAPK signaling pathways. Poult. Sci..

[20] Lv H., He Y., Wu J., Zhen L., Zheng Y. (2023). Chronic cold stress-induced myocardial injury: Effects on oxidative stress, inflammation and pyroptosis. J. Vet. Sci..

[21] Cain T., Brinsley J., Bennett H., Nelson M., Maher C., Singh B. (2025). Effects of cold-water immersion on health and wellbeing: A systematic review and meta-analysis. PLoS ONE.

[22] Son M., Wang A.G., Keisham B., Tay S. (2023). Processing stimulus dynamics by the NF-κB network in single cells. Exp. Mol. Med..

[23] Ye T., Tao W.-Y., Chen X.-Y., Jiang C., Di B., Xu L.-L. (2023). Mechanisms of NLRP3 inflammasome activation and the development of peptide inhibitors. Cytokine Growth Factor. Rev..

[24] Liu Y., Pan R., Ouyang Y., Gu W., Xiao T., Yang H., Tang L., Wang H., Xiang B., Chen P. (2024). Pyroptosis in health and disease: Mechanisms, regulation and clinical perspective. Signal Transduct. Target. Ther..

[25] Liu Y., Xue N., Zhang B., Lv H., Li S. (2022). Cold Stress Induced Liver Injury of Mice through Activated NLRP3/Caspase-1/GSDMD Pyroptosis Signaling Pathway. Biomolecules.

[26] Teng T., Yang H., Xu T., Sun G., Song X., Bai G., Shi B. (2022). Activation of Inflammatory Networks in the Lungs Caused by Chronic Cold Stress Is Moderately Attenuated by Glucose Supplementation. Int. J. Mol. Sci..

[27] Li D., Ma W., Xiong M., Xie P., Feng Y., Liu D., Qiao Y., Shi C. (2022). Water Rewarming After Seawater Hypothermia Mitigates IL-1β in Both Intestinal Tissue and Blood. Ther. Hypothermia Temp. Manag..

[28] Lyu Y., Wang T., Huang S., Zhang Z. (2023). Mitochondrial Damage-Associated Molecular Patterns and Metabolism in the Regulation of Innate Immunity. J. Innate Immun..

[29] Yamada K.P., Kariya T., Aikawa T., Ishikawa K. (2021). Effects of Therapeutic Hypothermia on Normal and Ischemic Heart. Front. Cardiovasc. Med..

[30] Chen-Izu Y., Banyasz T., Shaw J.A., Izu L.T. (2024). The Heart is a Smart Pump: Mechanotransduction Mechanisms of the Frank-Starling Law and the Anrep Effect. Annu. Rev. Physiol..

[31] Chen K., Schenone A.L., Gheyath B., Borges N., Duggal A., Popović Z.B., Menon V. (2019). Impact of hypothermia on cardiac performance during targeted temperature management after cardiac arrest. Resuscitation.

[32] Thiele H., Hassager C. (2026). Cardiogenic Shock. N. Engl. J. Med..

[33] Mastoor Y., Murphy E., Roman B. (2025). Mechanisms of postischemic cardiac death and protection following myocardial injury. J. Clin. Investig..

[34] Liu Y., Wu Y., Zhu Y., Li Q., Peng X., Zhang Z., Liu L., Liu L., Li T. (2024). Role of Excessive Mitochondrial Fission in Seawater Immersion Aggravated Hemorrhagic Shock-Induced Cardiac Dysfunction and the Protective Effect of Mitochondrial Division Inhibitor-1. Antioxid. Redox Signal..

[35] Bader W., Steinacher C., Fischer H.T., Glueckert R., Schmutzhard J., Schrott-Fischer A. (2023). Effects of Therapeutic Hypothermia on Macrophages in Mouse Cochlea Explants. Int. J. Mol. Sci..

[36] Huang Y., Jiang W., Zhou R. (2024). DAMP sensing and sterile inflammation: Intracellular, intercellular and inter-organ pathways. Nat. Rev. Immunol..

[37] Murao A., Aziz M., Wang H., Brenner M., Wang P. (2021). Release mechanisms of major DAMPs. Apoptosis.

[38] Marchi S., Guilbaud E., Tait S.W.G., Yamazaki T., Galluzzi L. (2022). Mitochondrial control of inflammation. Nat. Rev. Immunol..

[39] Xiao W., Zhu Z., Yu Z., Pan Y., Xue Q., Zhou Y., Shi J. (2024). A composite patch loaded with 2-Deoxy Glucose facilitates cardiac recovery after myocardial infarction via attenuating local inflammatory response. Sci. Rep..

[40] Chen R., Zou J., Chen J., Zhong X., Kang R., Tang D. (2025). Pattern recognition receptors: Function, regulation and therapeutic potential. Signal Transduct. Target. Ther..

[41] Patten D.A., Wilkinson A.L., O’Keeffe A., Shetty S. (2021). Scavenger Receptors: Novel Roles in the Pathogenesis of Liver Inflammation and Cancer. Semin. Liver Dis..

[42] Sun H., Li Y., Zhang P., Xing H., Zhao S., Song Y., Wan D., Yu J. (2022). Targeting toll-like receptor 7/8 for immunotherapy: Recent advances and prospectives. Biomark. Res..

[43] Kelley N., Jeltema D., Duan Y., He Y. (2019). The NLRP3 Inflammasome: An Overview of Mechanisms of Activation and Regulation. Int. J. Mol. Sci..

[44] Otani K., Koyama R., Tsuyama J., Sakai S., Hase K., Shichita T. (2025). Zoledronic acid attenuates ischemic brain injury by promoting ETS2 and MSR1 expression. Int. Immunol..

[45] Sheng W., Ji G., Zhang L. (2022). Role of macrophage scavenger receptor MSR1 in the progression of non-alcoholic steatohepatitis. Front. Immunol..

[46] Toldo S., Abbate A. (2024). The role of the NLRP3 inflammasome and pyroptosis in cardiovascular diseases. Nat. Rev. Cardiol..

[47] Yu P., Zhang X., Liu N., Tang L., Peng C., Chen X. (2021). Pyroptosis: Mechanisms and diseases. Signal Transduct. Target. Ther..

[48] Xie W., Peng M., Liu Y., Zhang B., Yi L., Long Y. (2023). Simvastatin induces pyroptosis via ROS/caspase-1/GSDMD pathway in colon cancer. Cell Commun. Signal. CCS.

[49] Liu Z., Wang M., Wang X., Bu Q., Wang Q., Su W., Li L., Zhou H., Lu L. (2022). XBP1 deficiency promotes hepatocyte pyroptosis by impairing mitophagy to activate mtDNA-cGAS-STING signaling in macrophages during acute liver injury. Redox Biol..

[50] Saller B.S., Wöhrle S., Fischer L., Dufossez C., Ingerl I.L., Kessler S., Mateo-Tortola M., Gorka O., Lange F., Cheng Y. (2025). Acute suppression of mitochondrial ATP production prevents apoptosis and provides an essential signal for NLRP3 inflammasome activation. Immunity.

[51] Ma Z., Tang P., Dong W., Lu Y., Tan B., Zhou N., Hao J., Shen J., Hu Z. (2022). SIRT1 alleviates IL-1β induced nucleus pulposus cells pyroptosis via mitophagy in intervertebral disc degeneration. Int. Immunopharmacol..

[52] Liu H., Huang Y., Zhao Y., Kang G.J., Feng F., Wang X., Liu M., Shi G., Revelo X., Bernlohr D. (2023). Inflammatory Macrophage Interleukin-1β Mediates High-Fat Diet-Induced Heart Failure with Preserved Ejection Fraction. JACC Basic Transl. Sci..

[53] Yuan Y., Martsch P., Chen X., Martinez E., Li L., Song J., Poppenborg T., Bruns F., Kim J.H., Kamler M. (2025). Atrial cardiomyocyte-restricted cleavage of gasdermin D promotes atrial arrhythmogenesis. Eur. Heart J..

[54] Weindel C.G., Ellzey L.M., Martinez E.L., Watson R.O., Patrick K.L. (2023). Gasdermins gone wild: New roles for GSDMs in regulating cellular homeostasis. Trends Cell Biol..

